# Robotic surgeries for patients with colorectal cancer who have undergone abdominal procedures: Protocol for meta-analysis

**DOI:** 10.1097/MD.0000000000010396

**Published:** 2018-04-13

**Authors:** Ming Hu, Changfeng Miao, Xiaopeng Wang, Yuntao Ma

**Affiliations:** Department of General Surgery, Gansu Provincial People's Hospital, Lanzhou, China.

**Keywords:** colorectal cancer, meta-analysis, previous abdominal surgery, robotic surgery

## Abstract

**Background:**

Although the safety and the advantages of laparoscopic and robotic colorectal surgeries have been confirmed, the use of both modalities in patients with previous abdominal surgeries (PAS) history remains uncertain. Herein, we perform a meta-analysis to investigate the impact of PAS on perioperative recovery outcomes from laparoscopic and robotic colorectal surgeries.

**Methods:**

We will search PUBMED, the Cochrane Library, the Chinese Biomedical database (CBM), WanFang data, China National Knowledge Infrastructure (CNKI) up to January 2018. Studies will be screened by title, abstract, and full text independently and in duplicate. Studies that report the impact of PAS on perioperative recovery outcomes from laparoscopic and robotic colorectal surgeries will be eligible for inclusion. Outcome variables will be assessed included combined resection, conversion, operation time, blood loss, number of retrieved lymph nodes, days to soft diet intake, length of hospital stay, and postoperative complications. Assessment of risk of bias and data synthesis will be performed using STATA SE 12.0. Heterogeneity among studies will be assessed using the *I*^2^ statistic.

**Results:**

Randomized controlled trials, prospective cohort studies, and propensity-matched comparative studies will be used for the quantitative synthesis of the meta-analysis to evaluate the impact of PAS on perioperative recovery outcomes from laparoscopic and robotic colorectal surgeries.

**Conclusions:**

We aim to draw an objective conclusion of the comparisons in aspects of perioperative outcomes and provide physicians level I evidences for clinical decision makings.

## 1 Introduction

Compared with open surgery, laparoscopy surgery has yielded better recovery outcomes and similar oncologic outcomes in the management of colorectal cancer.^[1,2]^ Science the application of robotic surgery in the early 2000s, its safety, efficacy, and cost-effectiveness were evaluated with lots of controversial.^[3,4]^

In general surgeries, abdominal adhesions form surgery procedures may affect the surgical outcomes.^[5]^ It is difficult and dangerous for patients with postoperative adhesions to gain a safe access to the peritoneal cavity by using a Veress blindly.^[6]^ Also, it may be impossible to place trocars in the appropriate locations.^[7]^ If the adhesions covered the planned trocar sites, it would increase the overall operation time.^[8]^ What is worse, severe adhesions may distort normal anatomical structures.^[9]^ During the laparoscopic or robotic surgeries, limited vision and reduced haptic sensation may hinder the surgeons in their attempt to overcome the inherent limitations caused by previous adhesions.^[10]^

Several studies have reported that a history of previous abdominal surgery (PAS) does not affect the clinical outcomes in laparoscopic colorrctal cancer.^[11]^ while other studies have reported that patients with PAS are more likely to have prolonged operation time, significantly higher incidence of open conversion, an inadvertent enterotomy, or postoperative ileus than patients without PAS.^[12]^ Although the safety and the advantages of laparoscopic and robotic colorectal surgeries have been confirmed, the use of both modalities in patients with a PAS history remains uncertain. Herein, we perform a meta-analysis to investigate the impact of PAS on perioperative recovery outcomes from laparoscopic and robotic colorectal surgeries.

## 2 Methods and analysis

This protocol for meta-analysis is performed according to the Preferred Reporting Items for Systematic Review and Meta-analysis Protocols (PRISMA-P) statement.^[13]^

## 3 Selection of studies

Eligibility criteria: included studies were selected according to the eligibility criteria which was listed as following: *population*: patients with colorectal cancer who have had a previous abdominal surgery; *invention*: robotic colorectal surgery; *comparator*: laparoscopy colorectal surgery; *outcomes*: combined resection, conversion, operation time, blood loss, number of retrieved lymph nodes, days to soft diet intake, length of hospital stay, and postoperative complications.

*Exclusion criteria*: Non-peer-reviewed articles, review articles, case reports, case series, animal studies, meeting abstracts, letters to the editor, commentaries, editorials, proceedings, nonpropensity-matched comparative studies, and other nonrelevant studies will be excluded from analysis.

## 4 Study design

Randomized controlled trials (RCTs), prospective cohort studies, and propensity-matched comparative studies will be used for the qualitative and quantitative synthesis of the meta-analysis.

## 5 Search strategy

We will perform a systematic literature search through January 2018 using PUBMED, the Cochrane Library, the Chinese Biomedical database (CBM), WanFang data, and China National Knowledge Infrastructure (CNKI) for relevant articles published in any language.

The relevant searching terms will match Medical Subject Heading terms, and the searches will be repeated immediately before the final analyses to identify additional studies for inclusion. An example of the PubMed search strategy is shown in Table 1.

**Table 1:**
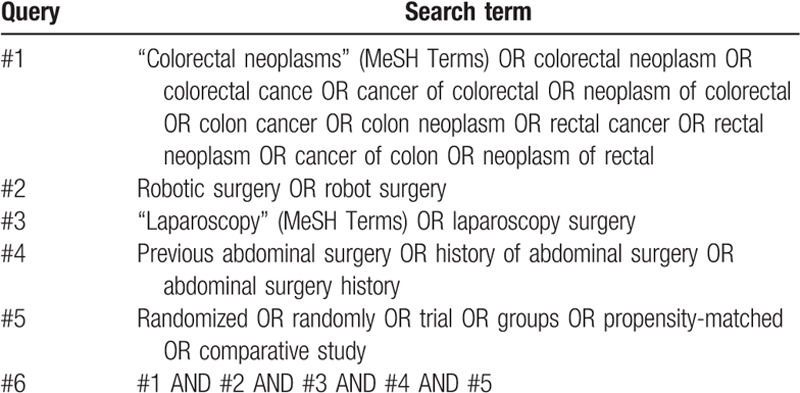
Search strategy for PubMed.

## 6 Data extraction

Data will be extracted from the included studies by 3 authors independently and recorded on a predesigned data collection form. We will extract the following study characteristics:

(1) *Study characteristics*: study design, number of study centers and locations, study setting, withdrawals, total duration of the trial, periods of data collection, follow-up duration.
(2) *Population characteristics*: inclusion and exclusion criteria, number, mean age, age range, gender, diagnostic criteria, pathological confirmation, staging of the tumor, TNM classification, and type of surgical procedure.
(3) *Intervention characteristics*: preoperative preparation, anesthetic protocol, and postoperative care.
(4) *Outcomes*: combined resection, conversion, operation time, blood loss, number of retrieved lymph nodes, days to soft diet intake, length of hospital stay, and postoperative complications.

## 7 Risk of bias assessment

Two investigators will independently assess the quality of included studies according to the following components, as advised in the Cochrane Handbook for Systematic Reviews of Interventions^[14]^: the method of random sequence generation; the method of allocation concealment; the methods of blinding of participants, researchers, and outcome assessors; the number of the participants lost to follow-up in each arm and the reasons for losses; whether all participants are analyzed according to their originally randomized group, that is, intention-to-treat (ITT) analysis; whether there are other problems that can put the study at high risk of bias, like baseline imbalance, deviation from the study protocol, dropouts or withdrawals from treatment, or insensitive outcome measurement tools; and selective reporting of outcomes. Each potential source of bias will be graded as high, low, or unclear and a quote from the study report with a justification for our judgement will be provided in the “Risk of bias” table. The risk of bias judgements across different studies for each of the domains listed will be summarized. We resolved disagreements by discussion with a third investigator. We contacted the trialists to seek clarification where necessary.

## 8 Data synthesis

Data from studies judged to be clinically homogeneous will be pooled using STATA SE 12.0 software. Heterogeneity between studies will be assessed using the Cochran's Q and Higgins *I*^2^ statistic. *P<*.10 for the Chi^2^ statistic or an *I*^2^ > 50% will be considered as showing considerable heterogeneity, and the data will be analyzed using the random-effect model. Otherwise, the fixed-effect model will be used. The Mantel–Haenszel method will be applied for pooling of dichotomous data and results will be presented as relative risk (RR) with their 95% confidence intervals (CI). Inverse variance method will be used for pooling of continuous data and results will be presented as standardized mean difference (SMD) with their 95% CI. *P* < .05 will be considered significant. If data are sufficient, we will conduct subgroup analyses between different surgical procedures: open surgery and minimally invasive surgery. Subgroup analyses will also be performed to explore potential sources of heterogeneity. Egger's regression test will be performed to assess the publication bias of the included studies. If there is a publication bias, trim and fill analysis will be performed.

## 9 Quality of evidence

We will evaluate the quality of evidence for the outcomes by using the Grading of Recommendations Assessment, Development and Evaluation (GRADE) system.^[15]^ The quality of evidence will be evaluated across the domains of risk of bias, consistency, directness, precision, and publication bias. According to GRADE, the quality of evidence can be rated as high, moderate, low, and very low, which is reflecting the strength of clinical recommendation.

## 10 Discussion

This protocol presents the methodology of a systematic review for assessing the feasibility, safety, and cost-effectiveness of robotic surgery for patients with colorectal cancer who have undergone abdominal procedures. We will comprehensively search, screen, assess, and extract valuable data from several databases as previously mentioned, and report this review results according to the PRISMA guidelines.

To our knowledge, this will be the first systematic review and meta-analysis using data of randomized controlled trials and propensity-matched comparative studies to compare the clinical outcomes between robotic and laparoscopic surgeries for patients with colorectal cancer who have undergone abdominal procedures updating to January 2018. The aim of our study is to draw an objective conclusion of the comparisons in aspects of perioperative outcomes and provide physicians level I evidences for clinical decision makings.

## Author contribution

MH and YTM participated in the study design, drafting the manuscript, and had significant role in development of the selection criteria, risk of bias assessment strategy, and data extraction criteria; MH and CFM participated in the study design and interpretation; CFM and XPW contributed to the development of the selection criteria, the risk of bias assessment strategy and data extraction criteria; MH and XPW developed the search strategy; MH and CFM participated in the statistical analysis. MH and YTM participated in critical review; all authors read, provided feedback, and approved the final manuscript.
